# Highly efficient *Agrobacterium rhizogenes*-mediated hairy root transformation for gene functional and gene editing analysis in soybean

**DOI:** 10.1186/s13007-021-00778-7

**Published:** 2021-07-10

**Authors:** Yuanyuan Cheng, Xiaoli Wang, Li Cao, Jing Ji, Tengfei Liu, Kaixuan Duan

**Affiliations:** Department of Plant Pathology, Nanjing Agricultural University, Nanjing, China

**Keywords:** Soybean, Hairy roots system, *Agrobacterium rhizogenes*, Gene function analysis, Gene editing

## Abstract

**Background:**

*Agrobacterium*-mediated genetic transformation is a widely used and efficient technique for gene functional research in crop breeding and plant biology. While in some plant species, including soybean, genetic transformation is still recalcitrant and time-consuming, hampering the high-throughput functional analysis of soybean genes. Thus we pursue to develop a rapid, simple, and highly efficient hairy root system induced by *Agrobacterium rhizogenes* (*A. rhizogenes*) to analyze soybean gene function.

**Results:**

In this report, a rapid, simple, and highly efficient hairy root transformation system for soybean was described. Only sixteen days were required for the whole workflow and the system was suitable for various soybean genotypes, with an average transformation frequency of 58–64%. Higher transformation frequency was observed when wounded cotyledons from 1-day-germination seeds were inoculated and co-cultivated with *A. rhizogenes* in 1/2 B5 (Gamborg’ B-5) medium. The addition of herbicide selection to root production medium increased the transformation frequency to 69%. To test the applicability of the hairy root system for gene functional analysis, we evaluated the protein expression and subcellular localization in transformed hairy roots. Transgenic hairy roots exhibited significantly increased GFP fluorescence and appropriate protein subcellular localization. Protein–protein interactions by BiFC (Bimolecular Fluorescent Complimentary) were also explored using the hairy root system. Fluorescence observations showed that protein interactions could be observed in the root cells. Additionally, hairy root transformation allowed soybean target sgRNA screening for CRISPR/Cas9 gene editing. Therefore, the protocol here enables high-throughput functional characterization of candidate genes in soybean.

**Conclusion:**

A rapid, simple, and highly efficient *A. rhizogenes*-mediated hairy root transformation system was established for soybean gene functional analysis, including protein expression, subcellular localization, protein–protein interactions and gene editing system evaluation.

**Supplementary Information:**

The online version contains supplementary material available at 10.1186/s13007-021-00778-7.

## Background

Soybean [*Glycine max* (L.)] is an important economic crop worldwide as a major source of oil and protein for humans and animals as well as for biofuel production [1]. A limited gene pool for some desirable traits and long-term breeding period are bottlenecks in soybean conventional breeding [2]. Soybean transformation has advantages over conventional breeding and plays a unique role in answering basic biological questions. Although soybean transformation systems have been developed and further improved over the past thirty years since they were first reported [3, 4], the stable genetic transformation of soybean is less efficient than in other crop species. This is particularly true that ever-increasing soybean genomics resources and especially genome editing technology needs to be best utilized to benefit soybean biotechnological applications and basic study. Therefore, a more efficient soybean transformation system for high-throughput functional characterization of genes is needed.

*Agrobacterium rhizogenes* (*A. rhizogenes*)-mediated hairy root transformation system is useful for species recalcitrant to transformation by *Agrobacterium tumefaciens* (*A. tumefaciens*) [5, 6]. Compared to *A. tumefaciens*-mediated transformation systems, *A. rhizogenes*-mediated hairy root transformation is more rapid and has a higher transformation frequency. Hairy root transformation systems have been established in soybean to study the metabolism, salt and drought stress, pathogens and nodulation [7–13]. However, the transformation frequency of current protocols is low and the process takes 30–45 days [14, 15]. This hampers the high-throughput characterization of gene function, emphasizing the need for a rapid, simple, and highly efficient procedure.

In this report, we describe a rapid, simple and highly efficient *A. rhizogenes*-mediated hairy root transformation system that enables the rapid characterization of gene function and gene editing in soybean. This system takes only 16 days for the whole workflow and has an applicability for various soybean genotypes. Transgenic hairy roots can be used for protein expression, subcellular localization, and bimolecular fluorescent complimentary (BiFC) analysis as well as target sgRNA screening for CRISPR/Cas9 gene editing. This simple and efficient soybean hairy root transformation method can also enable the investigation of root biology in plant species other than soybean.

## Methods

### Plant materials

Seeds of four soybean genotypes Williams 82, Zhonghuang 13, Magellan, and Maverick were used in this study. Seeds were surface sterilized for 16 h using chlorine gas produced by mixing 4 mL of 12 N HCl and 100 mL of commercial bleach (5.25% sodium hypochlorite) [16] in a tightly sealed desiccator.

### *Agrobacterium* strains and vector constructions

The binary plasmid pFGC5941 was used in this study. The *GUS* and *GFP* gene were ligated into pFGC5941 respectively, under the control of the cauliflower mosaic virus (CaMV) 35S promoter. The pFGC5941 vector contains *BAR* as a selection marker (conferring resistance to herbicide). For the localization experiment, GmSKRP1-fused GFP was ligated into pBIN, and mCherry-fused CAAX (a peptide sequence for membrane localization) was ligated into pFGC5941. For BiFC analysis, the full-length sequence of GmNSF (N-ethylmaleimide-sensitive factor) and GmSNAP (soluble NSF attachment protein) was PCR amplified and ligated into the pFGC5941-YFPN vector (C-terminal tag fused to YFPN) to yield pFGC5941-GmNSF-YFPN, and ligated into the pFGC5941-YFPC vector (C-terminal tag fused to YFPC) to yield pFGC5941-GmSNAP-YFPC. Next, the GmNSF-YFPN and GmSNAP-YFPC fragments were cloned and expressed separately with P2A peptide. For screening sgRNA, a codon-optimized cas9 gene with a nuclear localization sequence was assembled downstream of the CaMV 35S promoter together with an sgRNA driven by the U6 promoter. The sgRNA target sites have been reported previously [17].

*Agrobacterium rhizogenes* K599 [18] was used to induce hairy roots. *Agrobacterium tumefaciens* GV3101 was used in infiltration assays with *Nicotiana benthamiana*.

### *Agrobacterium rhizogenes*-mediated soybean hairy root transformation

A positive single colony of *A. rhizogenes* strain K599 was picked from the plate and transferred to 5 mL of liquid YEP (containing 50 mg/L kanamycin, 50 mg/L chloramphenicol, and 50 mg/L streptomycin). The mixture was subsequently incubated at 250 rpm for 12 h at 28 °C to get the starter culture. Next, 1 mL of the starter culture was transferred to 100 mL of YEP medium and incubated at 28 °C with shaking at 250 rpm until an optical density at 600 nm (OD600) of 1.2 was reached. Before inoculation, the cultures were centrifuged for 10 min at 3500 rpm and the pellets were resuspended in co-cultivation (CC) medium to an OD600 of 0.6. For CC medium evaluation, the liquid CC medium contained B5 (Sigma), 1/2 B5, or Murashige and Skoog (MS, Sigma), 1/2 MS salts supplemented with 0.5 g/L MES, 2% sucrose, 1.67 mg/L 6-BA, 0.25 mg/L GA3, and 40 mg/L acetosyringone (AS) (pH 5.4).

Using a scalpel we excised the 1/3 part of cotyledon with hypocotyl in each seed at an angle of 45 and transferred wounded cotyledons to agrobacterium CC medium for 30 min. After infection, the explants were evenly placed on solid CC medium and co-cultivated at 24℃ under a 16-h-light/8-h-dark photoperiod for 5 days. Solid CC medium contains B5 (Sigma), 1/2 B5 or MS (Sigma), 1/2 MS salts supplemented with 0.5 g/L MES, 2% sucrose, 0.7% agar, 1.67 mg/L 6-BA, 0.25 mg/L GA3, and 40 mg/L acetosyringone (AS) (pH 5.4). After co-cultivation, the explants were transferred to rooting medium (RM) and placed in an incubator at 24 °C in the dark for 10 days. The RM contains MS salts (Sigma) supplemented with 0.5 g/L MES, 2% sucrose, 200 mg/L cefotaxime (pH 5.7). We define 5 mm of regenerated root as a root because this is sufficient for DNA extraction and GUS staining.

### GUS histochemical staining and calculation of transformation frequency

GUS staining solution was prepared according to Jefferson's method [19]. Tissues were submerged in X-gluc solution overnight in the dark at 37°C and rinsed in 75% alcohol. Samples transformed with the empty vector pFGC5941 were used as negative controls. The transformation frequency (TF) was calculated as the percentage of roots with GUS expression in one cotyledon explant as follows: TF = (number of hairy roots with GUS expression per cotyledon explant ÷ total number of hairy roots per cotyledon explant) × 100%.

### Mutagenesis analysis with gene editing

Genomic DNA was extracted from transformed soybean hairy roots using the CTAB method [20]. To detect mutations, the target site was amplified by PCR using the *Glyma06g14180* forward primer (5′–GGAGCACTCCACCATCATCTAC-3′) and reverse primer (5′-GTTCTGACCTCAAA CCTTCAAA-3′) followed by digestion for 1.5 h with restriction enzyme *Pst*I. Products were detected using agarose gel electrophoresis. Undigested bands corresponding to targets were amplified, purified using a FastPure Gel DNA Extraction Mini kit (Vazyme Biotech, China), and ligated to a pMD20-T vector for sequencing. Clones were randomly selected and sequenced to detect gene mutations.

### *Agrobacterium tumefaciens* infiltration assays with *N. benthamiana*

A positive colony of *A. tumefaciens* GV3101 was picked and cultured in YEP medium with 50 mg/L kanamycin and 50 mg/L rifampicin for 12–16 h at 28 °C. The agrobacteria were collected and washed three times with infiltration buffer (10 mM magnesium chloride, 10 mM MES, and 150 mM acetosyringone [pH 5.6]) [21]. Next, the agrobacteria were resuspended in infiltration buffer and adjusted to an OD600 of 0.6. After inoculation, *N. benthamiana* leaves were incubated at 25 °C in the greenhouse for 2–3 days for next experiments.

### Confocal microscopy

Transgenic hairy roots and *N. benthamiana* leaves were prepared for fluorescence observations under an LSM 710 confocal laser scanning microscope (Carl Zeiss, Jena, Germany) with the following excitation wavelengths: GFP, 488 nm; RFP, 561 nm.

### Droplet digital PCR

The optimized droplet digital PCR (ddPCR) reaction mix (20 µL) contained 2 × QX200 ddPCR Supermix for Probes (Bio-Rad), 900 nM of forward and reverse primer (Additional file 1: Table S2), 250 nM of probe, and 25 ng of genomic DNA. Droplets were generated by the QX200™ Droplet Generator (Bio-Rad) and PCR was carried out in a C1000 Touch™ Thermal Cycler (Bio-Rad). PCR cycling conditions were as recommended by the manufacturer: 10 min of initial denaturation at 95 °C followed by 40 cycles of 30 s at 95 °C and extension for 1 min at 60 °C with a ramp speed of 2 °C/s; one cycle of 2 min at 90 °C and a hold at 20 °C. The fluorescence data were collected on the QX200 Droplet Reader (Bio-Rad). Samples with more than 15,000 total droplets and good separation between positive and negative droplets were selected for analysis.

### Statistical analysis

Each experiment was conducted at least three times independently with similar results. Statistical significance was determined using the unpaired two-sample *t*-test.

## Results

### A rapid and highly efficient strategy for *A. rhizogenes*-mediated hairy root transformation system in soybean

**Fig. 1:**
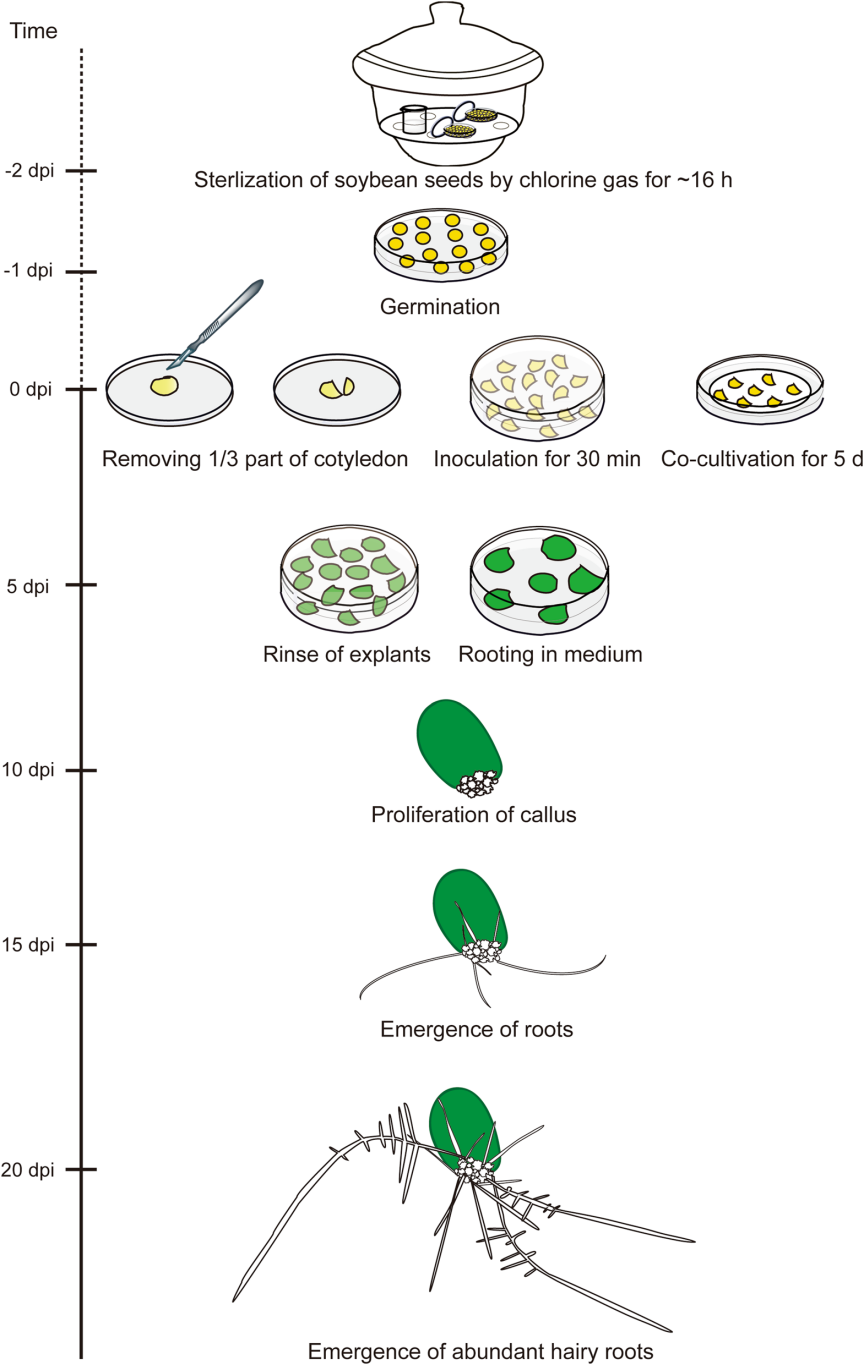
Workflow of the hairy root transformation for soybean by using cotyledon as explant

Our goal was to establish a rapid, simple and highly efficient *A. rhizogenes*-mediated hairy root transformation system in soybean. To this end, we substituted the soybean hypocotyl with cotyledon as explant for the *A. rhizogenes*-mediated hairy root transformation here (Fig. 1; see “Methods” for details). In brief, germinated seeds were excised the hypocotyl end with a sharp razor blade and only two-thirds of soybean cotyledons were left to increase the wounding surface for agrobacterium infection. Wounded cotyledons were inoculated with agrobacteria suspension for 30 min, followed by 5 days of co-cultivation (Fig. 1). Next, the infected cotyledons were rinsed to remove agrobacteria and transferred to RM to produce hairy roots. Hairy roots emerged at 15 days post-inoculation (Fig. 1). It took only 16 days from seed germination to starting to investigate gene function in the first batch of hairy roots.

**Fig. 2:**
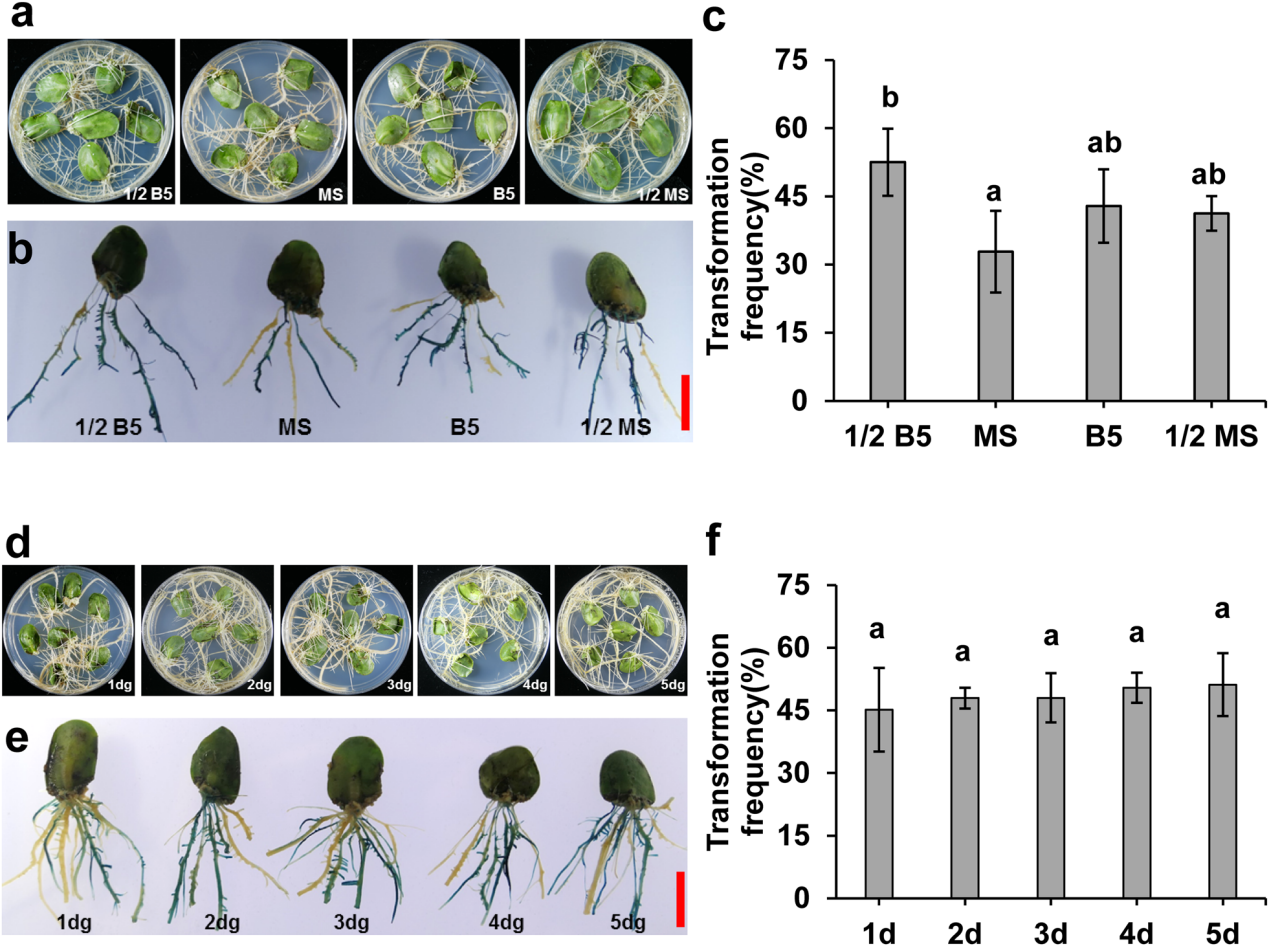
The effect of inoculation, co-cultivation medium and germination time of seed on hairy root transformation frequency. **a** Hairy root growth status after inoculated and co-cultivated with 1/2 B5, MS, B5, 1/2 MS medium. The photos were taken at 14 days post inoculation (dpi). Soybean genotype Williams 82 was used here. **b**, **e** GUS staining of transgenic hairy roots. **c**, **f** Positive hairy root transformation frequency (TF). **d** Hairy root growth status using cotyledons obtained from 1-, 2-, 3-, 4-, 5-day-germinated (dg) seeds as explants respectively. Soybean genotype Williams 82 was used here. Values are mean ± SD of three biological replicates, means with different letters denote a significance difference while similar letters denote no significance (Student’s t-test, P < 0.05). The scale bar indicates 1 cm

To optimize the transformation conditions, soybean genotype Williams 82 was used here. First, we established that higher efficient soybean transformation hairy root system was achieved when the cotyledons obtained from 1-day-germinated seeds were inoculated and co-cultivated with agrobacteria in 1/2 B5 medium. To evaluate the effect of different CC medium on transformation, we used 1/2 B5, MS, 1/2 MS, and B5 salts in CC liquid and solid media respectively. As shown in Fig. 2a, hairy root grew well after co-cultivation in 1/2 B5, MS, 1/2 MS and B5 medium. GUS staining showed that most hairy roots on each explant were positive transgenic roots (Fig. 2b). The negative control samples transformed with the empty vector pFGC5941 exhibited no GUS staining (Additional file 1: Fig. S1). Compared to the other three media, the highest transformation frequency of 53% (Fig. 2c) and longest hairy root length (Additional file 1: Fig. S2) was achieved with 1/2 B5 medium. Furthermore, the hairy root transformation frequency was not affected by use of cotyledons from 1-, 2-, 3-, 4-, 5-day-germinated seeds as explants (Fig. 2d–f). Therefore, cotyledons from 1-day-germinated seeds, and the 1/2 B5 co-cultivation medium were optimum and used in subsequent experiments.

**Fig. 3:**
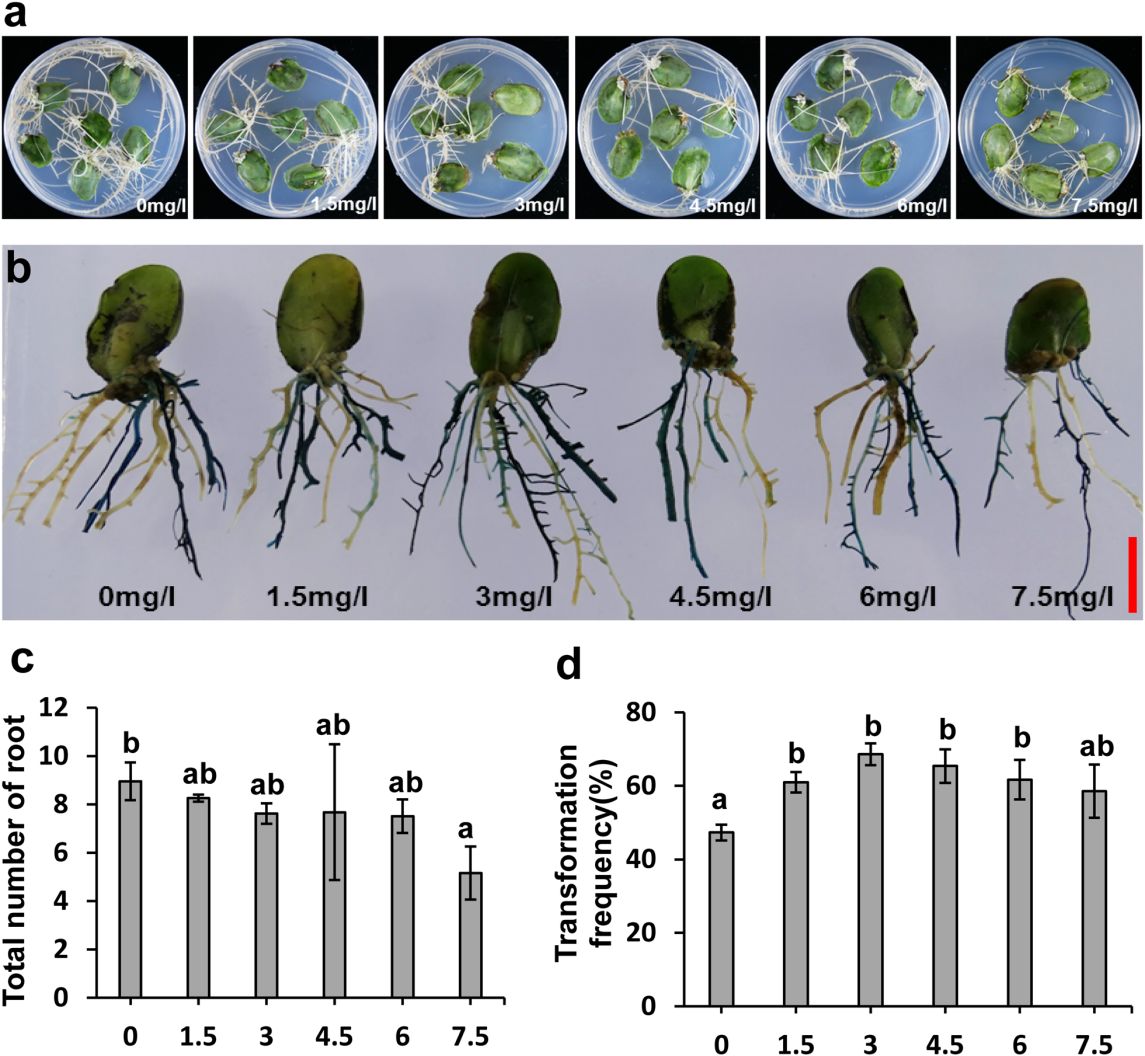
The effect of herbicide selection on hairy root growth and transformation frequency. **a** Hairy root growth status on MS medium with different concentration herbicide selection. Soybean genotype Williams 82 was used here. **b** GUS staining of transgenic hairy roots after growth on MS medium with different concentration herbicide selection. **c** Total number of hairy roots per explant on MS medium with different concentration herbicide selection. **d** Positive hairy root transformation frequency on MS medium with different concentration herbicide selection. Values are mean ± SD of three biological replicates, means with different letters denote a significance difference while similar letters denote no significance (Student’s t-test, P < 0.05). The scale bar indicates 1 cm

Second, the addition of herbicide selection to rooting medium increased the transformation frequency. The total number of hairy roots decreased as the herbicide concentration increased, indicating that herbicide selection inhibits non-transgenic hairy root growth (Fig. 3a, c). GUS staining revealed that selection with 3 mg/L herbicide in rooting medium yielded the highest transformation frequency of 69% (Fig. 3b, d). Therefore, cotyledons from 1-day-germinated seeds, 1/2 B5 inoculation and co-cultivation medium, and 3 mg/L herbicide selection were optimum for *A. rhizogenes*-mediated hairy root transformation.

**Fig. 4:**
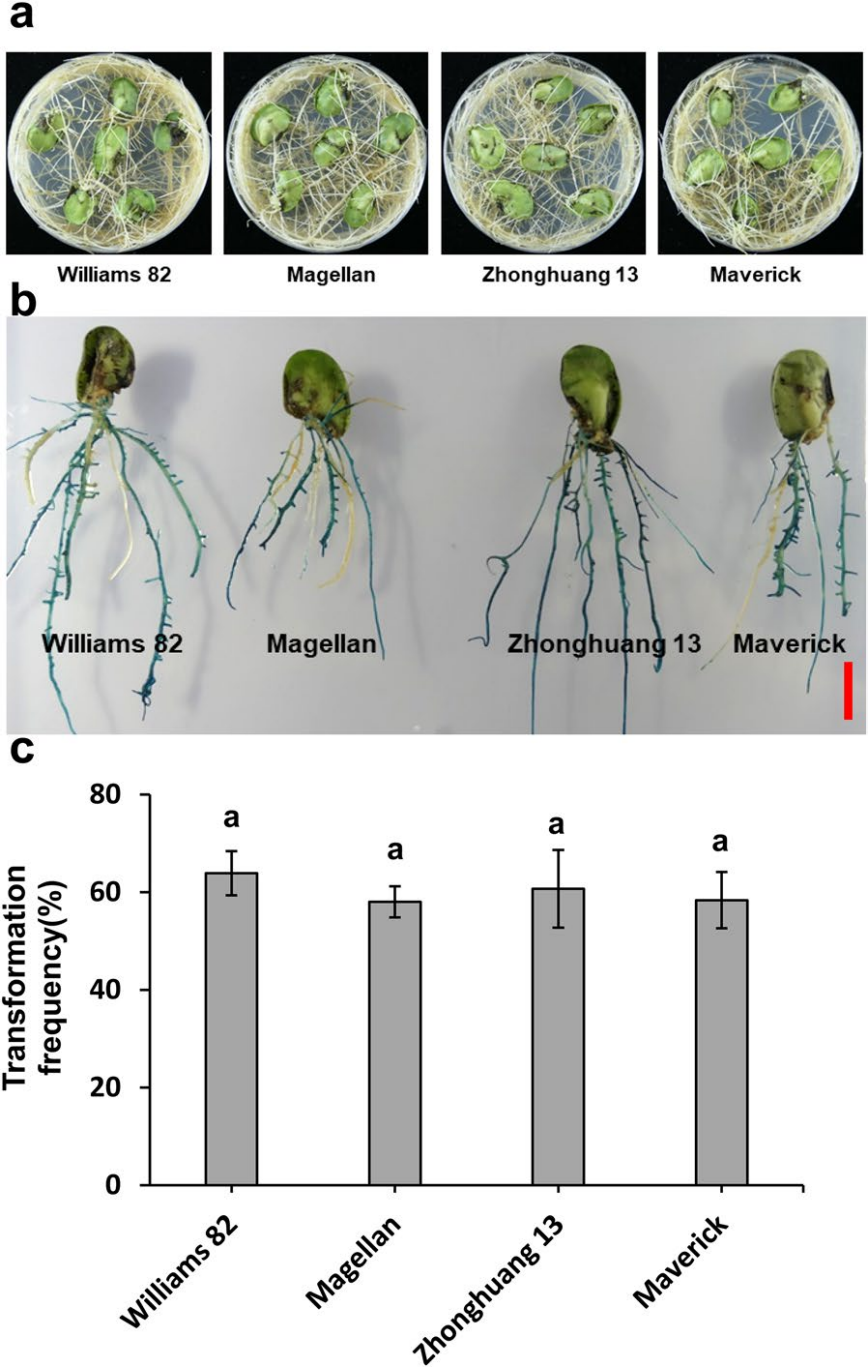
Hairy root transformation with different soybean genotypes.** a** Hairy root growth status from different soybean genotypes. **b** GUS staining of transgenic hairy roots from different soybean genotypes. **c** Positive hairy root transformation frequency from different soybean genotypes. Values are mean ± SD of three biological replicates, means with different letters denote a significance difference while similar letters denote no significance (Student’s t-test, P < 0.05). The scale bar indicates 1 cm

Moreover, we evaluated the hairy root transformation system in different soybean genotypes. There was no significant difference in transformation between the Williams82, Magellan, Zhonghuang13, and Maverick genotypes (Fig. 4), indicating broad applicability of the hairy root transformation system to various soybean genotypes. The system enables high-throughput functional characterization of candidate genes in soybean.

### Application of hairy root transformation for gene expression and protein subcellular localization in soybean

**Fig. 5:**
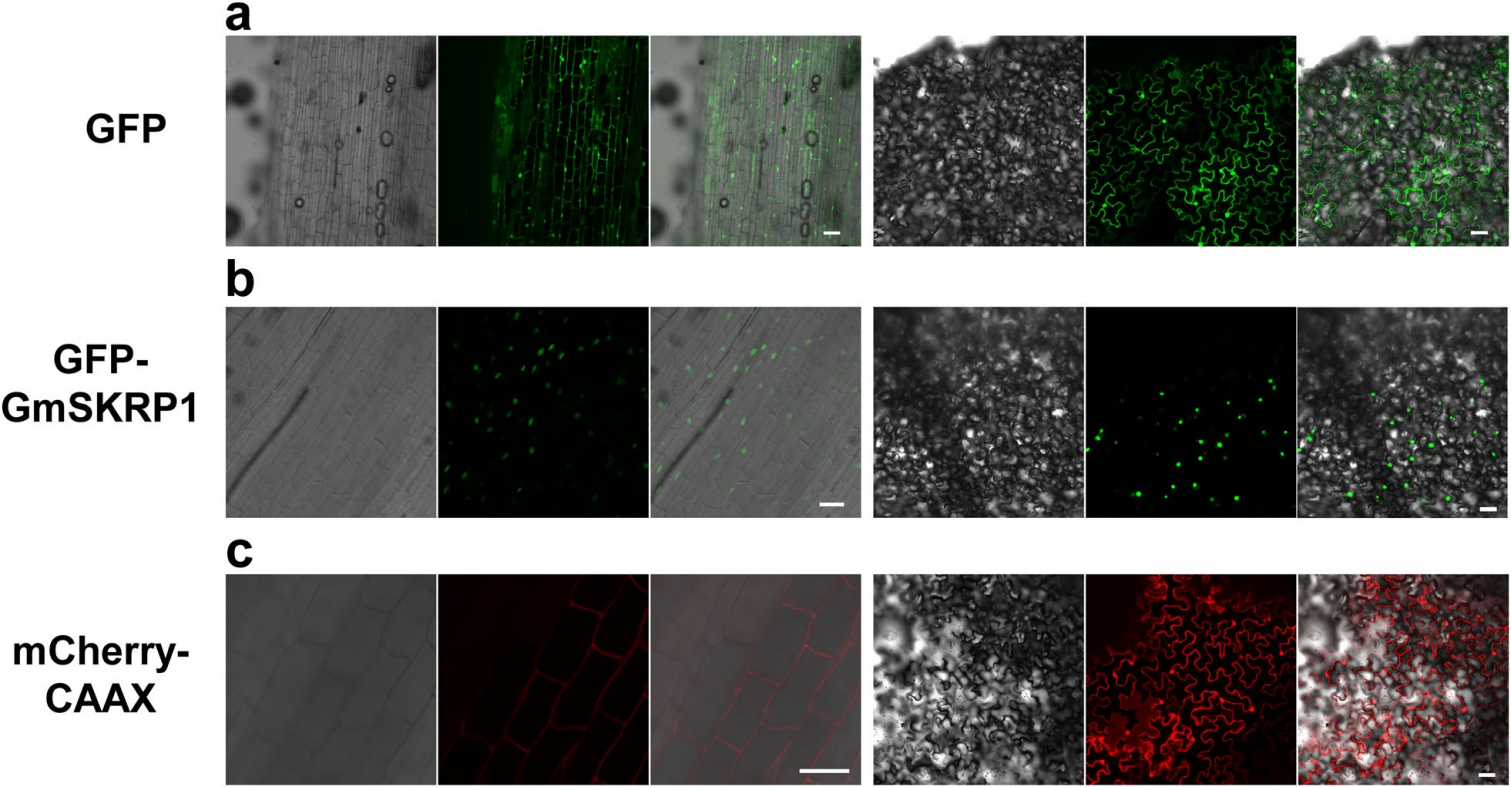
Protein subcellular localization with soybean hairy roots and tobacco leaves. **a** GFP expression and subcellular localization with soybean hairy roots (left) and tobacco leaves (right) respectively. Soybean genotype Williams 82 was used here. **b** GFP-GmSKRP1 fusion protein expression and subcellular localization with soybean hairy roots (left) and tobacco leaves (right) respectively. **c** mCherry-CAAX fusion protein expression and subcellular localization with soybean hairy roots (left) and tobacco leaves (right) respectively. Fluorescence was detected by confocal microscopy. The scale bar indicates 50 µm

Protein subcellular localization is important for charactering gene function. Plant protein subcellular localization is typically analyzed in *N*. *benthamiana* leaves by *Agrobacterium tumefaciens*-mediated transient transformation [21] because of the simplicity and high protein expression of this system. However, some organisms exhibit taxon-specific protein subcellular localization. Therefore, it is advantageous to analyze protein subcellular localization in soybean using soybean hairy roots instead of a system based on another species. We evaluated GFP expression, and nuclear and membrane protein localization in soybean hairy root cells. The results showed that GFP expression was high in transgenic soybean hairy roots and tobacco leaves (Fig. 5a). Soybean *GmSKRP1* (*Glyma.03G234200*) encoding a protein localized to the nucleus [22] was fused with GFP and transformed into soybean hairy roots and tobacco. As shown in Fig. 5b, GFP-GmSKRP1 exhibited nuclear localization in both soybean hairy root and tobacco leaf cells. Compared to tobacco, the cells of soybean hairy root could be observed much more clearly. mCherry-CAAX localized to cell membrane in soybean hairy roots, but to the membrane and nuclear in tobacco leaves (Fig. 5c). We inferred that protein overexpression in tobacco promoted nuclear localization. Therefore, the hairy root transformation system enables the evaluation of soybean protein expression and subcellular localization.

### Protein–protein interaction analysis with BiFC in soybean hairy root transformation system

**Fig. 6:**
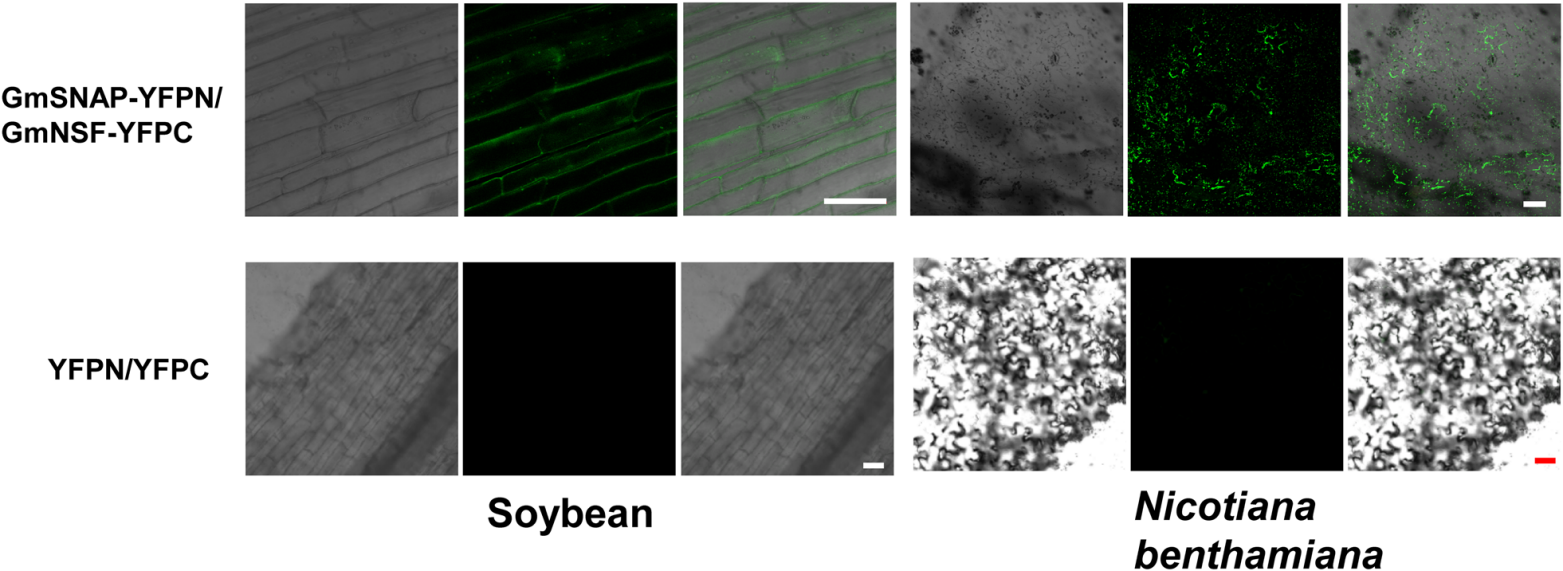
Protein–protein interaction by BiFC with soybean hairy roots and tobacco leaves.** a** YFPN-fused GmNSF and YFPC-fused GmSNAP were co-expressed in soybean hairy roots, and the co-expression of YFPN and YFPC was used as negative control. Soybean genotype Williams 82 was used here. **b** YFPN-fused GmNSF and YFPC-fused GmSNAP were co-expressed in tobacco leaves and the co-expression of YFPN and YFPC was used as negative control. Fluorescence was detected by confocal microscopy. The scale bar indicates 50 µm

To verify the protein–protein interactions, BiFC analysis was applied to in soybean hairy root cells. GmSNAP and GmNSF was selected to examine their interaction. GmSNAP [soluble NSF (N-ethylmaleimide-sensitive factor) attachment protein] and GmNSF were reported in sustaining cellular vesicle trafficking by mediating the disassembly and reuse of SNARE protein complexes, which facilitate the fusion of vesicles to target membranes [23]. YFPN-fused GmNSF and YFPC-fused GmSNAP were co-expressed in soybean hairy roots and tobacco leaves. As revealed in Fig. 6, we observed fluorescence spots indicating vesicle trafficking in hairy root and tobacco leaf cells co-expressing these two proteins, but not in the negative controls co-expressing only YFPN and YFPC (Fig. 6a, b), indicating that GmNSF and GmSNAP had an interaction with some vesicle trafficking characterization. However, there were some differences in expression levels in soybean hairy root and tobacco leaf cells. This means the GmNSF and GmSNAP protein–protein interaction may have special biological roles in its own organism and the hairy root system can help predict the actual functions of soybean genes more accurately.

### CRISPR/Cas9 gene-editing analysis with soybean hairy roots

**Fig. 7:**
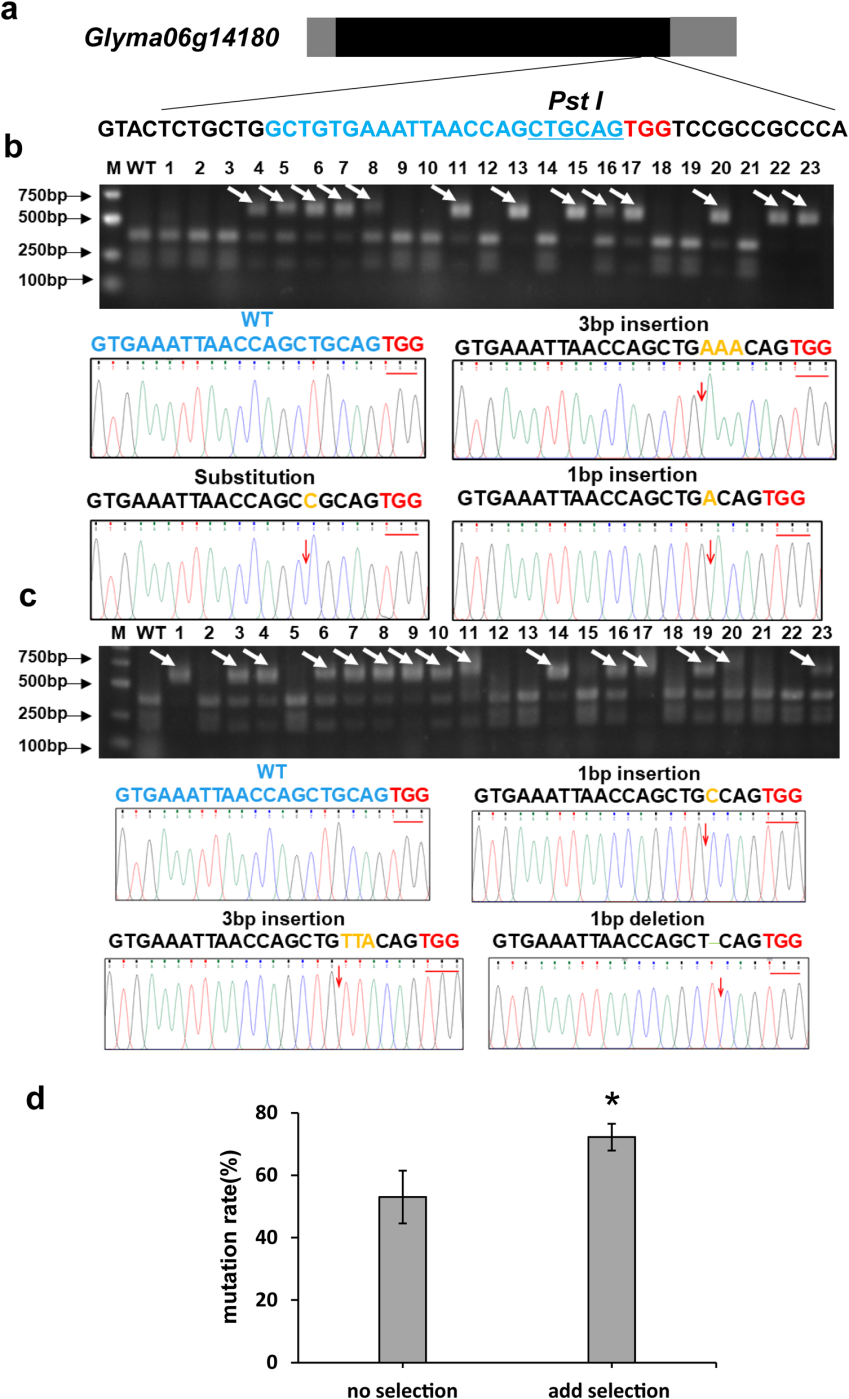
CRISPR-Cas9 gene editing with soybean hairy roots.** a** Gene structure and selected sgRNA target of soybean *Glyma06g14180.* Blue characters indicate sgRNA, Red characters indicate PAM sequence. Underline characters indicate *Pst I* enzyme site. **b**, **c** Electrophoresis and sequencing analysis of target gene editing. The target gene was amplified using gene specific primers and digested with the restriction enzyme (PCR-RE assay). Edited samples produced one large band (arrow indicates), whereas wild-type samples produced two small bands. Sequencing results showed different mutation type. Yellow characters indicate mutation sequence. **b** Showed results of samples from no herbicide selection rooting medium. **c** Showed results of samples from rooting medium with herbicide selection. **d** Editing efficiency of hairy roots from rooting medium with herbicide selection or no selection. Soybean genotype Williams 82 was used here. Values are mean ± SD of three biological replicates, means with different letters denote a significance difference while similar letters denote no significance (Student’s t-test, P < 0.05). *Indicates significant difference at 0.05 level by student’s t-test

In recent years, CRISPR/Cas9 system was well developed into a powerful gene editing tool [27, 28]. This technology accelerates crop improvement and biological research in various species, as well as in soybean [24–28]. However, due to the low frequency and time-consuming nature of soybean transformation, there are some risks to select low-editing-efficiency sgRNA targets when performing gene editing with CRISPR/Cas9 system. Therefore, it is necessary to screen high-editing-efficiency sgRNA targets before genetic transformation. Here we used the soybean hairy root system to verify gene editing efficiency and mutations. A plasmid harboring Cas9 and an sgRNA expression cassette was transformed into soybean cotyledons by *A. rhizogenes*. Fifteen days later, transgenic hairy roots were selected to investigate the gene editing efficiency. The target gene was amplified using gene-specific primers and digested with a restriction enzyme (PCR-RE assay) (Fig. 7a). The mutant gene was not digested because it had lost the enzyme site. As shown in Fig. 7b, after electrophoresis, the edited samples produced one large band, whereas wild-type samples produced two small bands. Sequence analysis revealed nucleotide substitutions, deletions, or insertions in the target gene, with an average editing efficiency of 53% (Fig. 7b). Furthermore, the editing efficiency was significantly higher in medium with a selective herbicide than in that without, with an average efficiency of 72% (Fig. 7c, d), suggesting that herbicide selection eliminated non-transgenic roots. Also, the hairy roots had different T-DNA copy numbers, indicating a single transformation event (Additional file 1: Table S1). Therefore, the soybean hairy root system is a powerful tool in high-throughput screening of sgRNA targets in CRISPR/Cas9 gene editing system.

## Discussion

### A rapid and highly efficient *Agrobacterium rhizogenes*-mediated hairy root transformation system in soybean

Current *A. tumefaciens*-mediated genetic transformation procedures of soybean is suboptimal for functional studies of soybean genes, because they are time-consuming, laborious, genotype-dependent, and hindered by low transformation frequency. Therefore, using *A. rhizogenes* to induce transformed hairy roots represents a novel approach in soybean gene functional analysis. Previous reports suggest that hairy roots can be induced from several soybean explant types, such as cotyledon and hypocotyl [14, 15]. These transgenic hairy roots have been used to evaluate salt and drought tolerance, nodulation, nematode and pathogen resistance, and virus-soybean interactions [7–13]. This was proven effective at gene functional analysis in soybean hairy root. According to the protocol reported previously, we think the procedure of soybean hairy root system still has potential to be optimized. Therefore, a faster, simpler and higher efficient soybean hairy root system is described here. In this report, we achieved a high hairy root transformation frequency by developing the transformation procedure. We select cotyledons obtained from 1-day-germinated seeds as explants. Wounded cotyledons inoculated and co-cultivated with *A. rhizogenes* in 1/2 B5 medium exhibited the highest positive transformation frequency of 53% (Fig. 2). The addition of a selective herbicide in root production medium increased the transformation frequency to 69% (Fig. 3) and the process takes only 16 days, faster than other methods [14, 15]. Unlike other protocols we used wounded cotyledons as explants, allowing agrobacteria infection better. In the co-cultivation medium, there are cytokinin (6-BA) and GA (GA3). These phytohormones promoted explants cell division and growth. For example, before root emergence, some calli emerged on the explants. Additionally, transferring explants from co-cultivation medium to RM directly enables the removal of agrobacterial contamination and the addition of herbicide to RM medium decreases the non-transgenic root emergence. This gives the explants better conditions for rooting. The protocol is also applicable to various soybean genotypes (Fig. 4), some of which are recalcitrant to shoot regeneration and transformation with *A. tumefaciens*. But in our hairy root system, there is no need to regenerate shoots, so the genotype has a lesser effect on the transformation efficiency. Nonetheless, a main limitation of this protocol is that it only allows the transformation of hairy roots, no other plant tissues. As a result, this can represent an advantage to investigate cell biology function of genes in roots other than in shoots.

### Application of soybean hairy roots transformation for protein subcellular localization and protein–protein interaction analyses

Protein subcellular localization and protein–protein interaction analyses are typically performed using *A. tumefaciens*-mediated transient transformation of tobacco leaves [21]. This system has the advantage of high protein expression. However, genes from other organisms may not express the true functional situation in tobacco leaves. As a result, using the hairy root transformation procedure here, we investigated the expression, and nuclear and membrane subcellular localization of soybean proteins. Transgenic hairy roots exhibited GFP and mCherry fluorescence as well as protein nuclear and membrane subcellular localization (Fig. 5). Compared to tobacco system, hairy roots had similar gene expression levels and the cells could be observed more clearly (Fig. 5), facilitating the characterization of gene function. The protein–protein interaction analysis showed different protein expression patterns of the GmNSF and GmSNAP interaction in soybean hairy root and tobacco leaf cells. The protein interaction in soybean hairy root exhibited a lower expression pattern but tobacco leaf exhibited a high expression pattern (Fig. 6). Therefore, the GmNSF and GmSNAP protein–protein interaction may have different biological functions in soybean and tobacco. Based on our results, the hairy root system is a powerful tool in soybean protein subcellular localization and protein–protein interaction. This system has potential for investigating soybean protein-DNA, and protein-RNA interactions.

### Applicability of hairy root system for validating sgRNA targets of CRISPR/Cas9 gene editing

So far, the CRISPR/Cas9 system was predominantly exploited for the generation of gene mutation, enabling the introduction of valuable novel traits in crops and phenotypic analysis of mutants in plant biology [24–30]. While the use of CRISPR/Cas9 system to generate new mutant is a time-consuming process, especially in crops such as soybean. Also, there is a risk of selecting inappropriate sgRNA targets, leading to failed or low-efficiency editing. As a result, profiting from the rapid and highly efficient transformation of hairy root, use of the CRISPR/Cas9 technology with hairy root transformation is a better choice to screening high-efficiency editing sgRNA targets before performing genetic transformation. Our results showed that the CRISPR/Cas9 system was able to edit soybean genes, with an editing efficiency of 72% when it was coupled with herbicide selection (Fig. 7). Because each hairy root represents a single transformation event (Table S1), the system allows high-throughput screening of sgRNA targets and modifying CRISPR/Cas9 systems, and thus opening a whole new range of applications of CRISPR/Cas9 gene editing in soybean.

## Conclusions

A rapid, simple, highly efficient and genotype-independent hairy root transformation system mediated by *A. rhizogenes* was established in soybean. The system allows high-throughput gene functional analyses and biotechnological applications in soybean, including protein expression, subcellular localization, protein–protein interaction, and gene editing. This method broadens the investigation of gene functions in species recalcitrant to transformation with *A. tumefaciens.*

## Supplementary Information

**Additional file 1: Figure S1** GUS staining of the negative control samples which were transformed with empty vector pFGC5941. **Figure S2** The hairy root length after inoculating in different media. **Table S1** Copy number of 35S in transgenic hairy root events estimated by ddPCR. **Table S2** Primers and probes used in droplet digital PCR.

## Data Availability

The datasets generated and analyzed in the current study are available from the corresponding author on reasonable request.
