# Supplementary material for: Highly efficient Agrobacterium rhizogenes-mediated hairy root transformation for gene functional and gene editing analysis in soybean
Source: Plant Methods. 2021 Jul 10;17:73. doi: 10.1186/s13007-021-00778-7 (PMC8272327; doi:10.1186/s13007-021-00778-7)
Supplement: Supplementary file 1 — Additional file 1: Figure S1 GUS staining of the negative control samples which were transformed with empty vector pFGC5941. Figure S2 The hairy root length after inoculating in different media. Table S1 Copy number of 35S in transgenic hairy root events estimated by ddPCR. Table S2 Primers and probes used in droplet digital PCR. [file 13007_2021_778_MOESM1_ESM.pptx]

## Slide 1
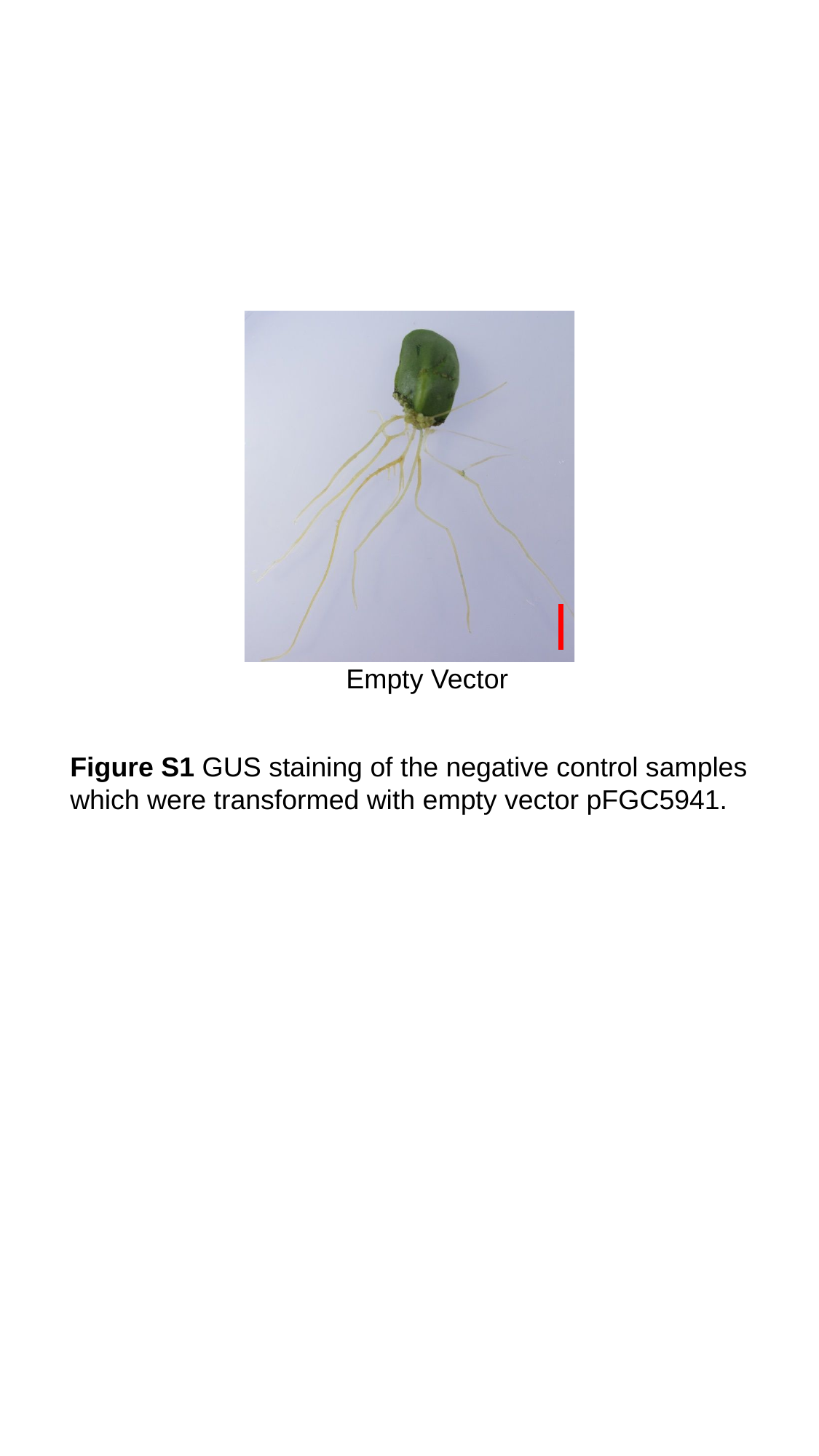

Empty Vector
Figure S1 GUS staining of the negative control samples which were transformed with empty vector pFGC5941.

## Slide 2
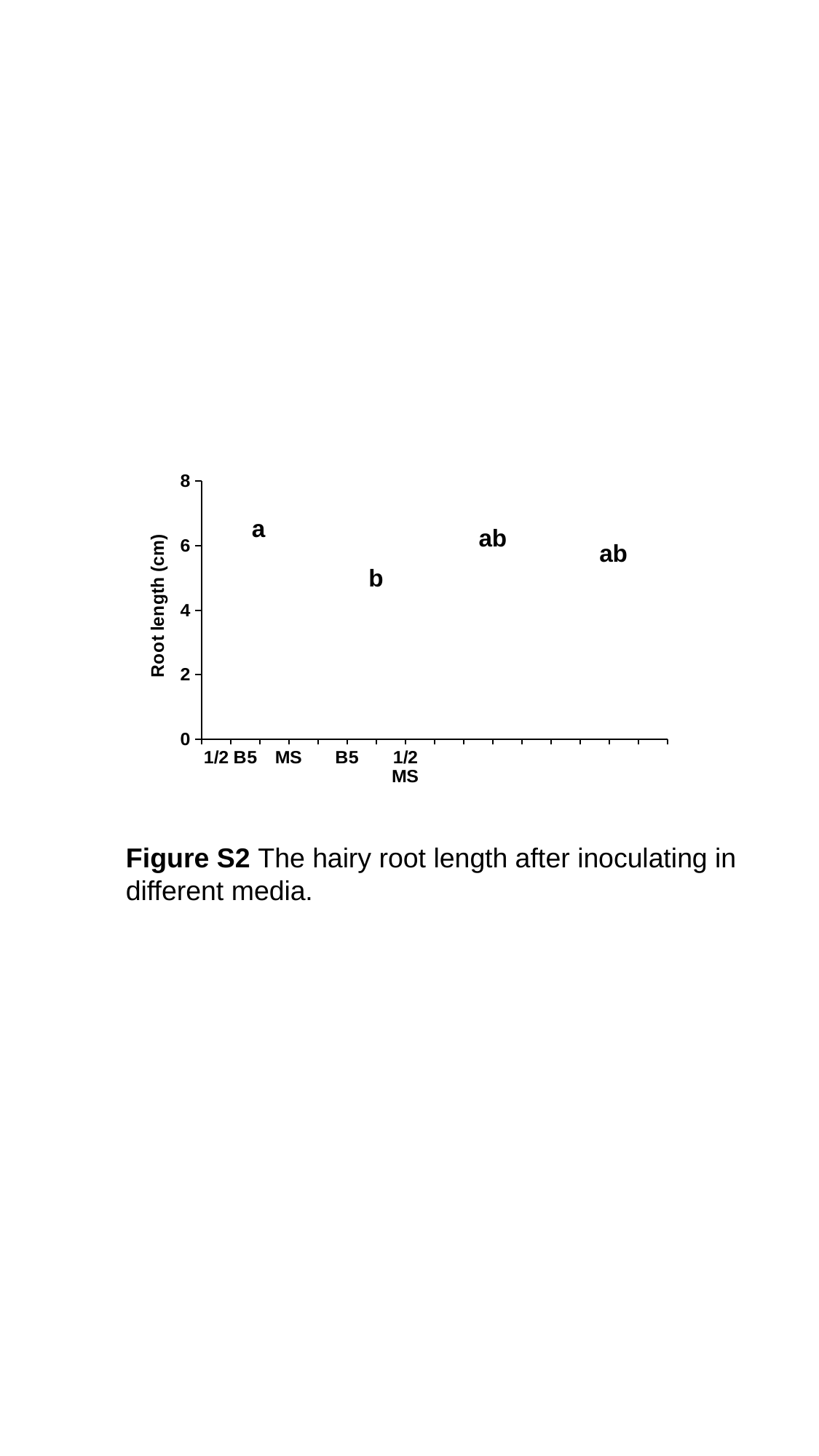

### Chart
| Category | |
|---|---|
| 1/2 B5 | 5.4143 |
| MS | 4.1012 |
| B5 | 5.1131 |
| 1/2 MS | 4.7155 |a
ab
ab
b
Figure S2 The hairy root length after inoculating in different media.

## Slide 3
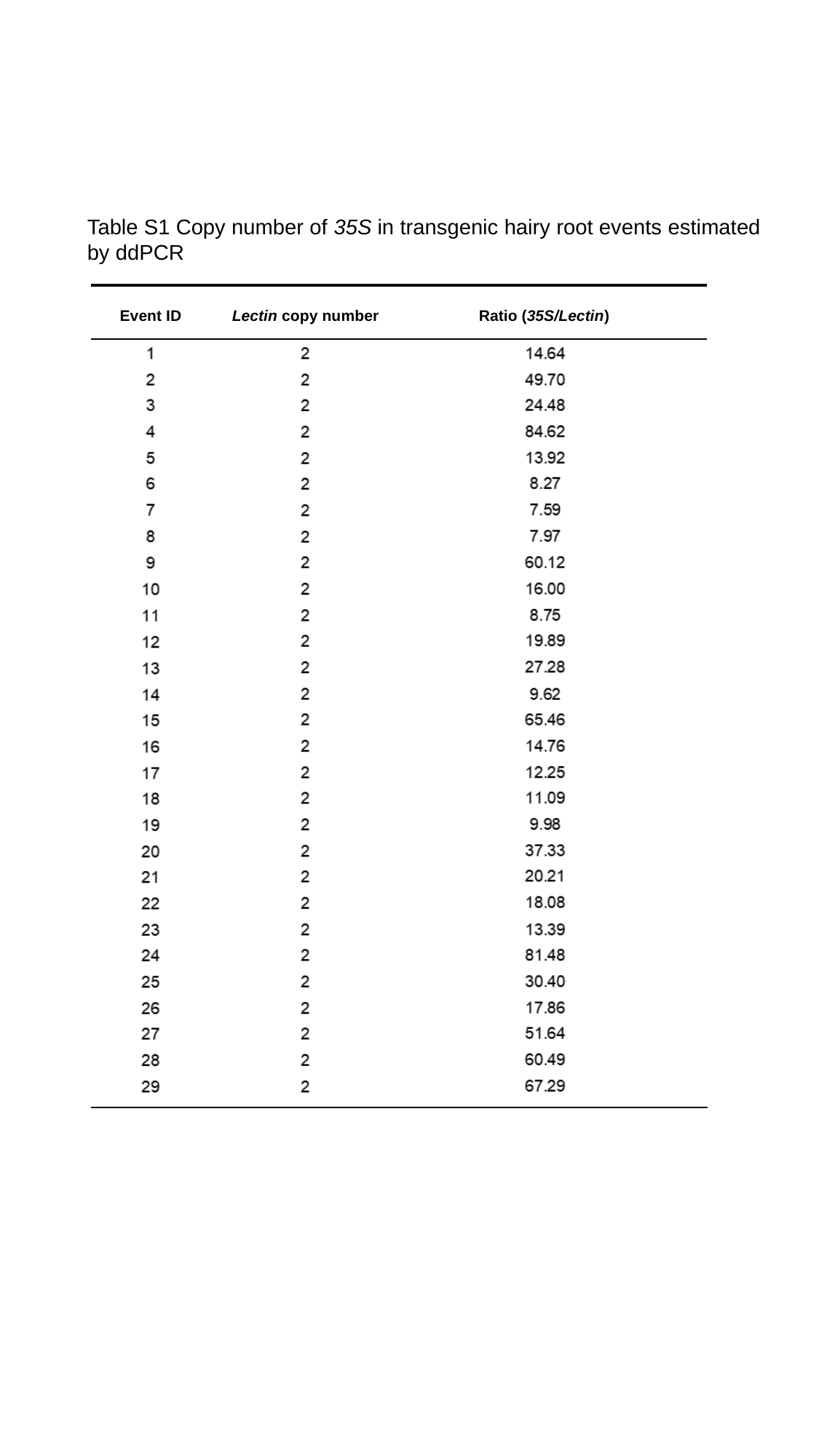

Table S1 Copy number of 35S in transgenic hairy root events estimated by ddPCR
Event ID
Ratio (35S/Lectin)
Lectin copy number

## Slide 4
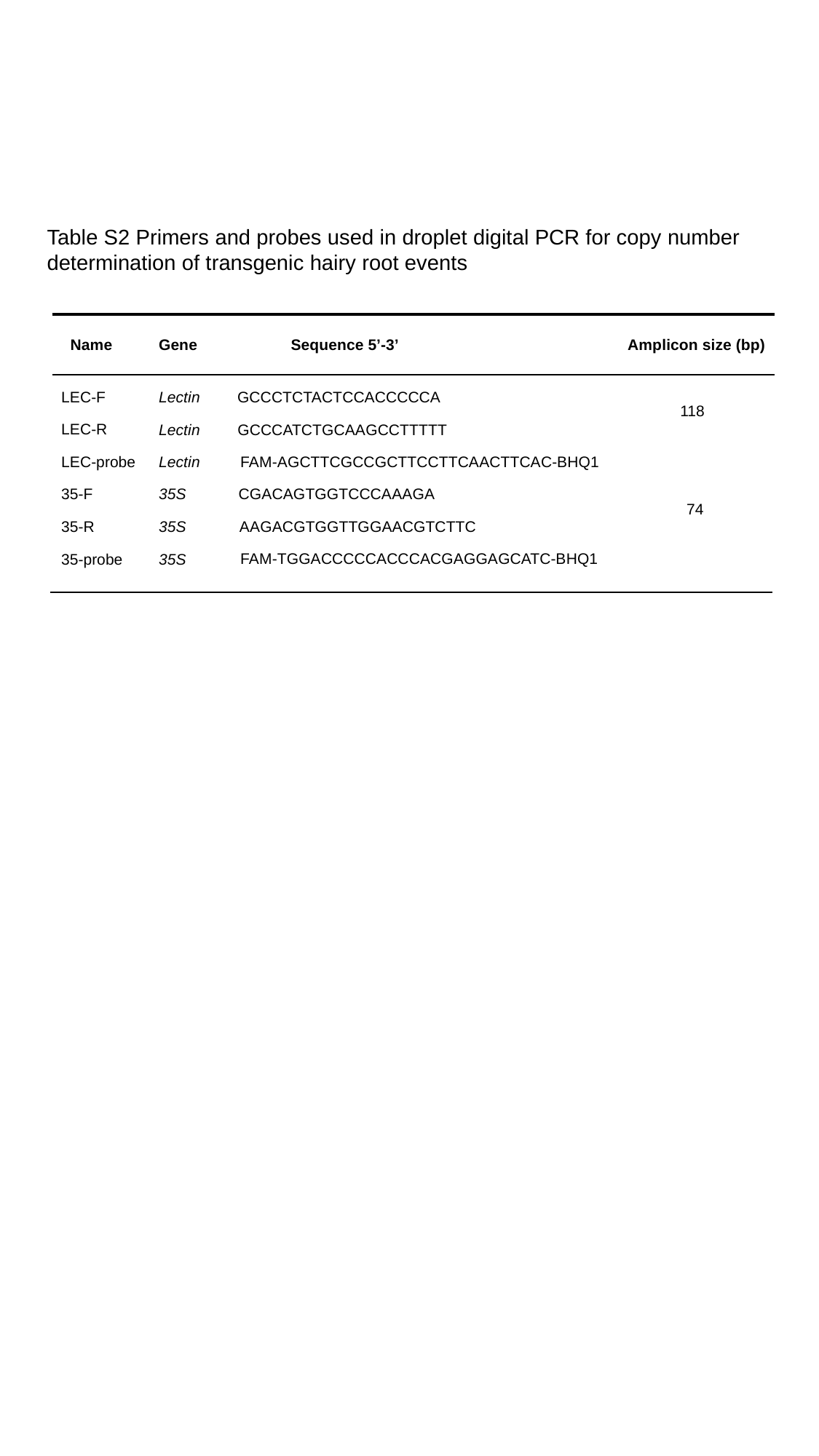

Table S2 Primers and probes used in droplet digital PCR for copy number determination of transgenic hairy root events
Name
Gene
Sequence 5’-3’
Amplicon size (bp)
LEC-F
Lectin
GCCCTCTACTCCACCCCCA
118
LEC-R
Lectin
GCCCATCTGCAAGCCTTTTT
LEC-probe
Lectin
FAM-AGCTTCGCCGCTTCCTTCAACTTCAC-BHQ1
35-F
35S
CGACAGTGGTCCCAAAGA
74
35-R
35S
AAGACGTGGTTGGAACGTCTTC
FAM-TGGACCCCCACCCACGAGGAGCATC-BHQ1
35-probe
35S
